# Impacto da Capacidade Funcional Pré-operatória nos Resultados Pós-Operatórios de Cirurgia de Cardiopatia Congênita: Estudo Observacional e Prospectivo

**DOI:** 10.36660/abc.20201137

**Published:** 2022-02-14

**Authors:** Angela Sachiko Inoue, Antônio Augusto Barbosa Lopes, Ana Cristina Sayuri Tanaka, Maria Ignêz Zanetti Feltrim, Filomena R.B.G. Galas, Juliano Pinheiro Almeida, Ludhmila Abrahão Hajjar, Emilia Nozawa

**Affiliations:** 1 Universidade de São Paulo Hospital das Clínicas São Paulo SP Brasil Universidade de São Paulo Hospital das Clínicas,São Paulo, SP – Brasil

**Keywords:** Cardiopatias Congênitas, Teste de Caminhada, Frequência Cardíaca, Desempenho Físico Funcional, Complicações Pós-Operatórias

## Abstract

**Fundamento:**

Apesar de avanços em técnicas cirúrgicas e cuidados pós-operatórios em cardiopatia congênita, a morbidade cardiovascular permanece elevada.

**Objetivo:**

Avaliar a associação do condicionamento pré-operatório de crianças e adolescentes com cardiopatias, mensurado por teste de caminhada de 6-minutos (TC6M) e variabilidade da frequência cardíaca (VFC), com a ocorrência de choque cardiogênico, séptico e morte no período pós-operatório.

**Métodos:**

Estudo clínico prospectivo e observacional de 81 pacientes de 8 a 18 anos. No período pré-operatório foram realizados o TC6M (distância caminhada e SpO_2_) e a VFC. O escore de risco ajustado para cirurgia de cardiopatia congênita ( *RACHS-1* ) foi aplicado para predizer o fator de risco cirúrgico para mortalidade. A ocorrência de pelo menos uma das complicações citadas foi considerada como evento combinado. Valores de p<0,05 foram considerados significantes.

**Resultados:**

Dos 81 pacientes, 59% eram do sexo masculino, com idade média de 12 anos; 33% eram cianóticos; e 72% já tinham realizado cirurgias prévias. O choque cardiogênico foi a complicação mais comum, e 31% apresentaram evento combinado. Cirurgia prévia, tipo de cardiopatia atual, *RACHS-1* , SpO_2_ em repouso, durante e após recuperação do TC6M foram selecionados para o estudo multivariado. A SpO_2_ após o TC6M permaneceu como fator de risco independente para aumentar a ocorrência de evento combinado no pós-operatório (OR: 0,93, IC95% [0,88 – 0,99], p=0,02).

**Conclusão:**

O SpO_2_ após o TC6M no período pré-operatório foi o fator independente preditor de prognóstico no pós-operatório em crianças e adolescentes submetidos à correção cirúrgica; a distância caminhada e as variáveis da VFC não tiveram a mesma associação.

## Introdução

Nas últimas décadas, pacientes com cardiopatias congênitas foram submetidos a cirurgias mais complexas, e apesar de avanços significativos nas técnicas cirúrgicas e cuidados pós-operatórios, a taxa de complicações ainda é alta, incluindo a morbidade cardiovascular.^1 , 2^ Os possíveis mecanismos por trás de desfechos pós-operatórios desfavoráveis nesses pacientes parecem ser o prejuízo ao desempenho funcional prévio, com capacidade aeróbica reduzida, associado à fraqueza muscular generalizada e a disfunções no sistema nervoso autônomo. Normalmente, a presença de algum dano cardíaco residual após a cirurgia pode ser parcialmente responsável pela capacidade física reduzida.^3^

Para avaliar a função cardiovascular geral, incluindo a capacidade física, alguns exercícios físicos são propostos para identificar fatores de risco para a ocorrência de eventos em várias situações clínicas, como a doença pulmonar obstrutiva crônica (DPOC)^4^ e insuficiência cardíaca.^5^ Muitos testes estão disponíveis para avaliar esta capacidade, mas seu uso em crianças e adolescentes pode gerar resultados diferentes daqueles obtidos entre adultos, devido a diferentes respostas fisiológicas e metabólicas ao estresse.^2 , 3 , 6^

Um dos métodos usados para a avaliação clinica da habilidade aeróbica é o teste de caminhada de 6 minutos (TC6M), que é um teste simples utilizado para verificar o nível de limitação funcional e a estratificação prognóstica em adultos e crianças.^7 , 8^ O teste foi usado para avaliar desfechos em diferentes estágios do tratamento de várias doenças, e demonstrou uma forte associação com a dessaturação da oxihemoglobina na cardiopatia crônica.

As anomalias na modulação do sistema nervoso autônomo, quando medidas pela variabilidade da frequência cardíaca (VFC), estiveram associadas ao aumento da mortalidade cardiovascular, ao pior prognóstico para doença cardíaca e aos eventos cardíacos pós-operatórios.^9^

O objetivo deste estudo foi avaliar a associação entre o status cardiovascular no período pré-operatório de crianças e adolescentes, medido pelo TC6M e pela VFC, e a ocorrência de choque séptico e cardiogênico, além de morte, no período pós-operatório de cirurgia cardíaca congênita.

## Métodos

### Desenho do estudo e pacientes

Este é um estudo clínico prospectivo e observacional conduzido de janeiro de 2009 a março de 2012, que inclui crianças e adolescentes de 8 a 18 anos com cardiopatia congênita, submetidos a tratamento cirúrgico de correção ou paliativo no Instituto do Coração do Hospital das Clínicas, Faculdade de Medicina da Universidade de São Paulo. Este estudo foi aprovado pelo Comitê de Ética em Pesquisa da Universidade de São Paulo, Faculdade de Medicina, Hospital das Clínica (número 0625/08). O termo de consentimento foi obtido para todos os pacientes, de seus pais ou tutores.

O estudo incluiu pacientes admitidos para o procedimento cirúrgico que estavam estáveis. Esses pacientes não estavam recebendo medicamentos inotrópicos ou vasoativos, não apresentavam nenhuma arritmia potencialmente séria ou complexa, como fibrilação atrial / ventricular, não tinham marca-passos, não apresentavam cardiomiopatia ou doença valvular adquirida, não tinham síndromes associadas nem limitações pulmonares, neurológicas ou ortopédicas. Pacientes que não forneceram o consentimento ou não foram submetidos ao procedimento cirúrgico foram excluídos.

### Coleta de dados

Os dados foram coletados de registros médicos durante o período de internação. Após a indicação para cirurgia, a avaliação da VFC e do TC6M foi realizada no mesmo dia, nesta ordem.

No período pré-operatório, as variáveis idade, gênero, índice de massa corporal (IMC), diagnóstico clínico (com maior impacto clínico) e a ocorrência de cirurgias prévias foram coletadas. O escore de risco ajustado para cirurgias para cardiopatia congênita (RACHS-1)^10^ também foi aplicado para predizer o fator de risco cirúrgico para mortalidade. Para o uso deste escore, casos de cirurgia cardíaca congênita são colocados de uma a seis categorias de risco, com base na presença ou ausência de alguns diagnósticos; a categoria 1 tinha o menor risco e, a 6, o maior risco. Em relação às variáveis intraoperatórias e de procedimento, os dados foram coletados com base no tipo de cirurgia, tempo de circulação extracorpórea, tempo de ventilação mecânica (VM), e uso de medicamentos vasoativos e/ou inotrópicos. No período pós-operatório, a ocorrência de morte e complicações até a alta do paciente foram analisadas. As complicações pós-operatórias consideradas no estudo foram: morte, choque cardiogênico (sangramento persistente, necessitando de transfusão de sangue, necessidade de oxigenação por membrana extracorpórea (ECMO)), suspeita de tamponamento cardíaco e reexploração cirúrgica, choque refratário, levando ao suporte inotrópico para manter a pressão arterial média de ≥ 60mmHg (por mais de 72 horas), parada cardiorrespiratória, arritmias significativas (incluindo fibrilação atrial, taquicardia ventricular, bloqueio atrioventricular) e choque séptico (confirmação do local da infecção e uso de antibióticos, confirmado por infecção, que gera febre persistente, insuficiência respiratória, requerendo VM prolongada, e uso de ventilação não-invasiva, hipotensão persistente e leucocitose ou leucopenia). A ocorrência de pelo menos uma das complicações listadas foi considerada como evento combinado.

### Teste de caminhada de 6 minutos

O TC6M foi realizado no período pré-operatório, de acordo com a técnica padrão proposta pela American Thoracic Society,^7^ em um corredor de 30 metros e uma só repetição. Além da distância percorrida, a frequência cardíaca, saturação de oxigênio (SpO_2_) (Ohmeda^®^), pressão arterial (Philips^®^ esfigmomanômetro digital), frequência respiratória, sensação subjetiva de dispneia e fadiga nos membros inferiores foram medidas com a escala modificada de Borg^11^ em repouso, imediatamente após o teste e três minutos depois da recuperação. Frases de incentivo padrão foram usadas a cada minuto durante o teste. Nenhum paciente precisou de oxigênio durante a atividade.

#### Variabilidade da frequência cardíaca

Um monitor cardíaco (Polar s810i^®^) foi utilizado e os sinais elétricos do coração foram transmitidos para um monitor por meio de uma cinta de eletrodos colocada em volta do peito do paciente. A VFC foi avaliada no pré-operatório, em repouso, por 15 minutos, com o paciente na cama em uma inclinação de 45 graus. Os cinco primeiros minutos foram usados para adaptação, e os últimos 10 minutos, para análise. O sinal foi recebido e enviado para o software Polar Precision Performance.^12^ O artefato foi removido com o mesmo software, e manualmente, por meio da inspeção visual dos intervalos R-R (oscilações nos intervalos entre as batidas consecutivas do coração) e da exclusão de intervalos anormais. As amostras que apresentavam mais de 85% de batidas sinusais foram incluídas. A análise de VFC utilizando métodos lineares foi realizada utilizando o Kubiot HRV, versão 2.0 (Biosignal Analysis and Medical Imaging Group). As variáveis de tempo analisadas foram a SDNN (desvio padrão de todos os intervalos RR normais gravados em um intervalo de tempo); rMSSD (raiz quadrada média das diferenças sucessivas); e pNN50 (porcentagem das diferenças sucessivas entre os intervalos RR que são maiores que 50 ms). As variáveis analisadas no domínio frequência (análise de espectro) foram o componente da alta frequência (AF) (0,15 a 0,4 Hz); o componente de baixa frequência (BF) (0,04 e 0,15Hz); a razão AF/BF; e a normalização dos dados da análise de espectro para minimizar os efeitos de outras bandas, como um componente de frequência muito baixa. As variáveis SDNN, rMSSD, pNN50 e AF estiveram associadas à modulação parassimpática, enquanto a BF esteve associada tanto à modulação simpática quanto à parassimpática.^13^

#### Análise estatística

As variáveis quantitativas com distribuição normal foram apresentadas como média e desvio padrão. As variáveis quantitativas sem distribuição normal foram apresentadas como mediana e intervalo interquartil (IIQ); percentis 25 e 75). As variáveis categóricas são apresentadas como taxas de frequência e porcentagem. O teste de Shapiro-Wilk foi usado para avaliar a distribuição das variáveis quantitativas. O cálculo do tamanho da amostra se baseou em uma amostra piloto em pacientes no período pré-operatório; para estudar o prognóstico por meio de testes com 90% de poder e 5% de nível de significância, a predição mínima foi de 75 casos. Para a análise univariada, com relação ao evento combinado, o teste t de Student não-pareado ou o teste de Mann-Whitney foram usados para variáveis quantitativas; e o teste de qui-quadrado ou o teste de razão de verossimilhança foram usados para variáveis categóricas. Para a análise multivariada, as variáveis com p<0.10 foram usadas no modelo de regressão logística múltipla para avaliar fatores prognósticos de morte e morbidade. O valor de p de probabilidade <0.05 foi usado como critério de significância estatística. Todas as análises foram realizadas com o SPSS 15.0 para Windows.

## Resultados

**Figura 1:**
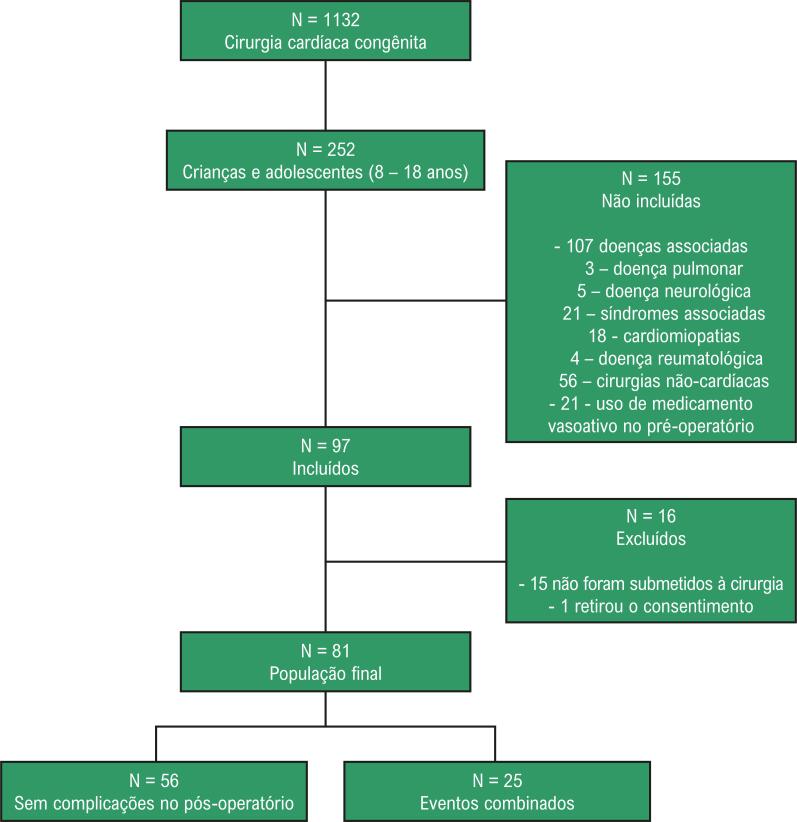
– Fluxograma do estudo.

Noventa e sete crianças e adolescentes foram avaliados, dos quais 15 não foram submetidos à cirurgia, e um interrompeu o protocolo, retirando o consentimento, sendo assim excluídos do estudo ( Figura 1 ). Dos 81 pacientes do estudo, 59% eram do sexo masculino, com idade média de 12 anos; 33% estavam cianóticos; e 72% tinham realizado cirurgia cardíaca previamente. O choque cardiogênico foi a complicação mais frequente, e 31% tiveram evento combinado com a ocorrência de pelo menos uma das outras complicações. A mortalidade neste estudo foi de 6,2% ( Tabela 1 ).

**Tabela 1:** – Complicações no pós-operatório de crianças e adolescents submetidos à cirurgia cardíaca congênita

Complicações no pós-operatório	n (%)
Choque cardíaco	24 (29,6)
Choque séptico	5 (6,2)
Morte	5 (6,2)
Evento combinado	25 (30,9)

Os fatores: RACHS-1 e SpO_2_ de recuperação estiveram significativamente associados à ocorrência de desfechos combinados e estão apresentados na Tabela 2 . No período pós-operatório, os tempos de circulação extracorpórea, VM, estada na UTI, alta do hospital e procedimento cirúrgico estiveram significativamente associados à ocorrência de desfechos combinados ( Tabela 3 ).

**Tabela 2:** – Valores descritivos das variáveis pré-operatórias de acordo com o grupo de ocorrência de evento combinado em crianças e adolescentes submetidos à cirurgia cardíaca congênita

Variável		Evento combinado		p

	Geral	Não	Sim	

	n=81	n=56 (69,1%)	n=25 (30,9%)	
Idade (anos)^*^	12 ± 3	12 ± 3	13 ± 2	0,190
Sexo^†^				0,204
Feminino	31 (37,5%)	24 (42,9%)	7 (28,0%)	
Masculino	50 (62,5%)	32 (57,1%)	18 (72,0%)	
IMC (kg/m^2^)^Δ^	17 (12 – 27)	17(15 - 21)	17 (15 - 20)	0,581
Cirurgia prévia^†^				0,098
Não	23 (28%)	19 (33,9%)	4 (16,0%)	
Sim	58 (72%)	37 (66,1%)	21 (84,0%)	
N – cirurgias prévias^‡^				0,227
0	23 (28,40%)	19 (33,9%)	4 (16,0%)	
1	25 (30,87%)	18 (32,1%)	7 (28,0%)	
2	29 (35,81%)	17 (30,4%)	12 (48,0%)	
3	4 (4,92%)	2 (3,6%)	2 (8,0%)	
Doença cardíaca atual^Ϯ^				0,089
Acianótico	53 (65,43%)	40 (71,4%)	13 (52,0%)	
Cianótico	28 (34,57%)	16 (28,6%)	12 (48,0%)	
Rachs-1^‡^				0,018
1	6 (7,41%)	6 (10,8%)	0 (0,0%)	
2	22 (27,16%)	18 (32,1%)	4 (16,0%)	
3	53 (65,43%)	32 (57,1%)	21 (84,0%)	
Distância caminhada (metros)*	521 ± 99	517 ± 89	502 ± 94	0,480
FC em repouso (bpm)*	86 ± 14	87 ± 15	84 ± 10	0,326
FC – TC6M (bpm)*	127 ± 17	123 ± 20	127 ± 22	0,423
FC - recuperação (bpm)*	92 ± 14	92 ± 14	90 ± 11	0,372
SpO_2_ em repouso (%)^Δ^	96 (84 – 97)	96 (85 - 98)	89 (80 - 97)	0,076
SpO_2_ – TC6M (%)^Δ^	94 (74 – 91)	94 (74 - 97)	83 (65 - 96)	0,050
SpO_2_ – recuperação (%)^Δ^	96 (85 – 97)	97 (87 - 97)	87 (81 - 96)	0,009
SDNN (ms) ^Δ^	37 (23 – 59)	37 (26 - 50)	33 (22 - 41)	0,184
rMSSD (ms) ^Δ^	26 (13 – 46)	29 (14 - 46)	20 (14 - 28)	0,157
pNN50 (%)^Δ^	3,8 (0,2 – 26)	7,3 (0,3 – 25,6)	2.2 (0.3 – 5.8)	0,222
BF (Hz) ^Δ^	402 (86 – 816)	318 (148 - 812)	312 (94 - 445)	0,225
AF (Hz) ^Δ^	216 (46 – 479)	229 (39 - 526)	103 (46 - 254)	0,189
BFn.u (%)^Δ^	35 ( 50 – 79)	66 (50 - 80)	63 (53 - 80)	0,971
AFn.u (%)^Δ^	35 (21 – 55)	34 (21 - 52)	37 (20 - 47)	0,905
BF/AF ^Δ^	2 (1 – 3,9)	2 (1 – 3,9)	1,7 (1,1 - 4)	0,967

*: t de Student não-pareado; ^†^: teste de qui-quadrado; ^Δ^: Mann-Whitney; ^‡^: teste de verossimilhança; n(%): número (%), ou média±desvio padrão ou mediana (interval interquartil); IMC: índice de Massa Corporal; Rachs 1:escore de risco ajustado para cirurgias para cardiopatia congênita; FC: frequência cardíaca; bpm: batidas por minuto; SpO_2_: saturação periférica de oxigênio; TC6M: teste de caminhada de 6 minutos; SDNN: desvio padrão de todos os intervalos RR normais gravados em um intervalo de tempo; rMSSD: raiz quadrada média das diferenças sucessivas; pNN50: porcentagem das diferenças sucessivas entre os intervalos RR que são maiores que 50 ms; BF: componente de baixa frequência variando de 0,04 a 0,15Hz; AF: componente de alta frequência variando entre 0,15 e 0,4 Hz; n. u.: unidade de normalidade.

**Tabela 3:** – Valores descritivos de variáveis pós-operatórias de acordo com o grupo de ocorrência de eventos combinados em crianças e adolescentes submetidos à cirurgia cardíaca congênita

Variável		Evento combinado		p

	Geral	Não	Sim	

	n = 81	n=56 (69,1%)	n=25 (30,9%)	
Tempo de circulação extracorpórea (min)*	110 ± 70	92 ± 57	156 ± 76	<0,001
Tempo de VM (horas)^Δ^	11 ± 20	5.5 (3,3 - 8)	13,8 (7,8 – 19,2)	<0,001
Estada na UTI (dia)^Δ^	8 ± 5	6 (4 - 8)	10 (7 - 16)	0,001
Alta do hospital (dia)^Δ^	17 ± 12	11 (8 - 17)	21 (14 - 27)	0,001
Procedimento cirúrgico^†^				0,023
Correção septal atrial	6 (7,4%)	6 (10,7%)	0 (0,0%)	
Correção septal ventricular	7 (8,6%)	5 (8,9%)	2 (8,0%)	
Tubo VD-AP	13 (16,1%)	7 (12,5%)	6 (24,0%)	
Procedimento de Fontan	16 (19,8%)	9 (16,1%)	7 (28,0%)	
Substituição da válvula mitral	1 (1,2%)	0 (0,0%)	1 (4,0%)	
Substituição da válvula tricúspide	2 (2,5%)	2 (3,6%)	0 (0,0%)	
Procedimento de Glenn	2 (2,5%)	1 (1,8%)	1 (4,0%)	
Istmoplastia	9 (11,1%)	9 (16,1%)	0 (0,0%)	
Ligadura do canal arterial	1 (1,2%)	1 (1,8%)	0 (0,0%)	
Dilatação da artéria pulmonar	6 (7,4%)	5 (8,9%)	1 (4,0%)	
Substituição da válvula pulmonar	3 (3,7%)	1 (1,8%)	2 (8,0%)	
Correção da TGA	2 (2,5%)	1 (1,8%)	1 (4,0%)	
Correção da tetralogia de Fallot	2 (2,5%)	0 (0,0%)	2 (8,0%)	
Correção de Ebstein	3 (3,7%)	2 (3,6%)	1 (4,0%)	
Cirurgia de Blalock-Taussig	1 (1,2%)	1 (1,8%)	0 (0,0%)	
Correção anômala das veias pulmonares	1 (1,2%)	1 (1,8%)	0 (0,0%)	
Substituição da válvula aórtica	4 (4,9%)	4 (7,1%)	0 (0,0%)	
Outros	2 (2,5%)	1 (1,8%)	1 (4,0%)	

*: t de Student não-pareado; ^Δ^: Mann-Whitney; ^†^: teste do qui-quadrado; n(%): número (%), ou média±desvio padrão ou mediana (intervalo interquartil); VM: ventilação mecânica; UTI: unidade de terapia intensiva; VD-AP: tubo entre o ventrículo direito e a artéria pulmonar; TGA: transposição das grandes artérias.

Quando os valores de SpO2 foram divididos em grupos de doença cardíaca cianótica e acianótica, os grupos demonstraram diferença significativa em relação ao SpO_2_ em repouso, o TC6M e a recuperação. O grupo acianótico apresenta valores significativamente maiores de SpO_2_ em comparação ao grupo cianótico ( Tabela 4 ).

**Tabela 4:** – Valores de SpO2 na doença cardíaca cianótica e acianótica em diferentes momentos do TC6M

Variável	Doença cardíaca atual		p ^Δ^

	Acianótica	Cianótica	

	n=53	n=28	
SpO_2_ em repouso (%)^Δ^	97 (93 - 98)	78 (75 -83)	<0,001
SpO_2_ – TC6M (%)^Δ^	95 (96 - 97)	63 (56.5 – 68.8)	<0,001
SpO_2_ – em recuperação (%)^Δ^	97 (93 – 97.5)	80.5 (77 – 83.8)	<0,001

*^Δ^: Mann-Whitney; mediana (IIQ)*

(interval interquartil); IMC: índice de Massa Corporal; *Rachs* 1:escore de risco ajustado para cirurgias para cardiopatia congênita; FC: frequência cardíaca; bpm: batidas por minuto; SpO_2_: saturação periférica de oxigênio; TC6M: teste de caminhada de 6 minutos; SDNN: desvio padrão de todos os intervalos RR normais gravados em um intervalo de tempo; rMSSD: raiz quadrada média das diferenças sucessivas; pNN50: porcentagem das diferenças sucessivas entre os intervalos RR que são maiores que 50 ms; BF: componente de baixa frequência variando de 0,04 a 0,15Hz; AF: componente de alta frequência variando entre 0,15 e 0,4 Hz; n. u.: unidade de normalidade.

**Figura 2:**
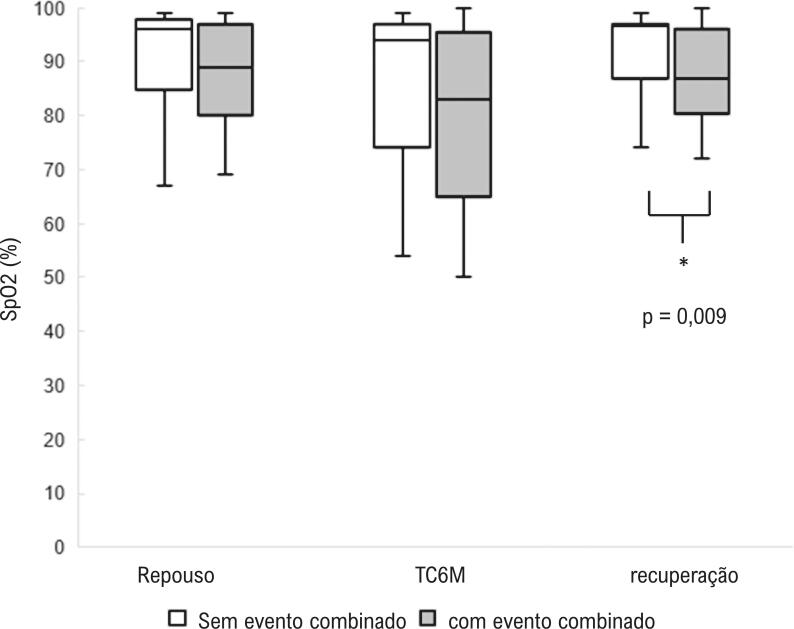
– Probabilidades estimadas pelo modelo de regressão logística para SpO_2_ de recuperação.

As variáveis prévias à cirurgia, tipos de doença cardíaca atual, RACHS-1, SpO_2_ em repouso no pré-operatório, durante o TC6M e na recuperação, e o tempo de circulação extracorpórea foram selecionados para compor a análise multivariada. O SpO2 no pré-operatório durante a recuperação manteve-se como um fator de risco independente (OR 0,93, IC95% [0.88 - 0.99], p = 0,02) para mais eventos combinados ( Figura 2 ).

Observou-se que quanto maior o SpO_2_ após o tempo de recuperação, menor a chance de ter eventos combinados. Por meio da curva ROC, observamos que o ponto de corte para SpO_2_ é 96% (OR 3,28, IC95% [1,21 – 8,90], p = 0,02). Este ponto demonstra 68% de sensibilidade, 60,7% de especificidade e 63,0% de precisão. As chances de um paciente com SpO_2_ menor que 96% na recuperação apresentar eventos combinados é três vezes maior do que para pacientes com SpO_2_ maior que 96%.

**Figura 3:**
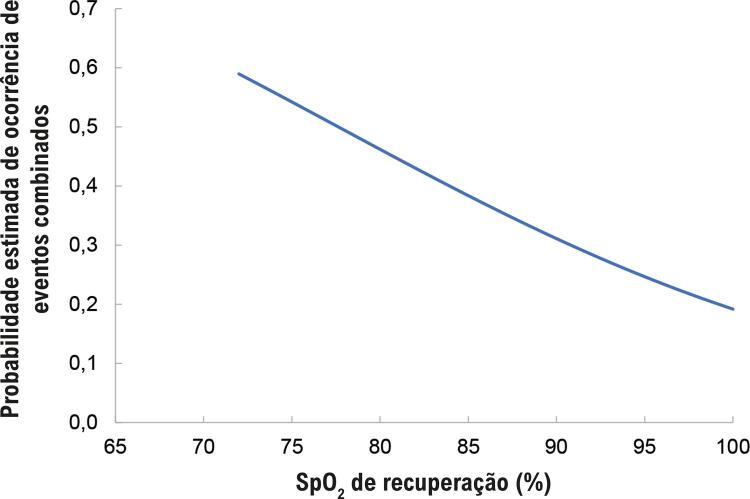
– Box-plot com comparação da saturação de oxigênio no TC6M em repouso, durante o teste e na recuperação, em grupos com e sem eventos combinados.

Observamos que na comparação entre os valores de SpO_2_ nos três momentos (em repouso, no TC6M e na recuperação), em grupos com e sem um evento combinado, SpO_2_ foi mais baixo, com diferença estatisticamente significativa, no período de recuperação no grupo de eventos combinados ( Figura 3 ).

## Discussão

Este estudo identificou que a variável SpO_2_ no período pré-operatório após o TC6M foi o único preditor independente da associação com a ocorrência de complicações pós-operatórias em crianças e adolescentes submetidos à correção cirúrgica de cardiopatia congênita, e que a VFC não foi capaz de prever a mesma associação. Esses dados sugerem que medir o SpO_2_ pode ser uma ferramenta importante para o prognóstico pós-operatório. Ao observar os valores baixos de SpO_2_ no pré-operatório, ações clínicas podem ser tomadas para otimizar a função cardiorrespiratória e, assim, possivelmente reduzir os eventos combinados nesta população de pacientes.

Em crianças, o TC6M foi o teste de escolha em relação aos testes cardiorrespiratórios em esteiras ou bicicletas ergométricas, já que é fácil de aplicar, é seguro e econômico, já que não requer equipamentos caros nem profissionais altamente qualificados. Estudos apontam que a falta de incentivo e a superproteção em torno desta população de pacientes têm impactos negativos em sua capacidade física e aumentam o risco de desenvolver complicações com o passar do tempo.

A avaliação da distância caminhada apresentou valores menores do que os apresentados por Geiger et al.^14^ e Priesnitz et al.^15^ em crianças saudáveis com mais de 8 anos de idade, indicando que crianças com cardiopatia congênita têm menos capacidade física do que aquelas da população saudável. Porém, este estudo observou que a distância média caminhada foi semelhante entre pacientes com eventos combinados e aqueles que progrediram sem complicações.

Monitorar a oximetria de pulso durante o TC6M não é um procedimento padrão; porém, pode oferecer uma estimativa melhor da troca de gases durante o exercício, mostrando, assim, uma melhor correlação com o prognóstico. O mecanismo da dessaturação de oxigênio pode estar diretamente relacionado ao defeito cardíaco que leva à maior resistência vascular, sobrecarga ventricular, principalmente no ventrículo direito, resultando em débito cardíaco diminuído. A dessaturação de oxigênio durante o TC6M é bem descrita em pacientes com DPOC^4 , 16^ e doença pulmonar intersticial,^17 , 18^ mas esses dados, até onde sabemos, não são bem descritos entre crianças para determinar o prognóstico no período pós-operatório, e este estudo pode incentivar a realização de novas análises.

Schaan et al.^19^ avaliaram a capacidade funcional de crianças e adolescentes com cardiopatia congênita em uma revisão sistemática e meta-análise, e descobriram que o consumo máximo de oxigênio (VO_2max_) foi a variável associada à baixa capacidade funcional, possivelmente sendo influenciada pela resposta cronotrópica defasada. Nenhuma medida da oximetria do pulso foi reportada nos estudos apresentados.

Essas mudanças anatômicas e fisiopatológicas podem estar associadas à resposta cronotrópica diminuída nesta população de pacientes. A necessidade de reintervenção é frequente e, consequentemente, as chances de dessensibilização do receptor adrenérgico beta podem estar diretamente relacionadas à regulação autônoma alterada.^9 , 20^ Neste estudo, 72% dos pacientes já tinham sido submetidos à cirurgia cardíaca prévia; porém, não foram observadas diferenças significativas entre os pacientes que apresentavam ou não complicações pós-operatórias.

Em um estudo de Hami et al.,^21^ no qual a VFC foi avaliada em cirurgias que requeriam a atriotomia, não foi possível demonstrar a influência do procedimento cirúrgico associado à essa redução. Este estudo observou que o procedimento de Fontan foi o tipo de cirurgia mais frequente, em 19,8% dos casos avaliados. Estudos com esses pacientes mostraram um declínio na capacidade física com o tempo, atribuído à VFC reduzida.^22^ O uso de diferentes técnicas (atriopulmonar com túnel lateral ou extracardíaco) para este procedimento não parece interferir, em princípio, com a redução da VFC. Porém, a técnica usando o tubo extracardíaco parece preservar o nó sinoatrial, reduzindo o risco de arritmias. Embora a técnica de fenestração tenha reduzido a ocorrência de complicações pós-operatórias,^23^ a redução do SpO_2_ permanece como ponto de preocupação, como identificado em nosso estudo.

A taxa de mortalidade neste estudo foi de 6,2%, corroborando outros estudos que mostraram uma incidência entre 3,6% e 15%. Aproximadamente 66% dos pacientes foram classificados na Categoria 3 de RACHS-1, que, de acordo com Jenkins et al.,^10^ tem uma taxa de mortalidade de aproximadamente 9,5%, confirmando a complexidade das doenças cardíacas. Todas as mortes que ocorreram na UTI se desenvolveram após a insuficiência cardíaca pós-operatória.

Finalmente, neste estudo, outras variáveis estiveram associadas com complicações pós-operatórias. Identificamos que os tempos de circulação extracorpórea, VM, estada na UTI, alta do hospital e procedimento cirúrgico estiveram significativamente associados à ocorrência dos desfechos combinados. Giamberti et al.^24^ descreveram que a morbidade grave é relativamente frequente, e normalmente associada às condições pré-operatórias (alto nível de hematócritos devido à cianose, insuficiência cardíaca congestiva, e número de operações prévios) e operatórias (procedimento de Fontan/conversão e duração da circulação extracorpórea) do paciente. Na verdade, 84% dos pacientes com complicações foram submetidos à cirurgia prévia e tinham mais tempo de circulação extracorpórea, VM e tempo de estada no hospital.

Nosso estudo tem limitações em potencial, como a inclusão de uma amostra heterogênea e a não inclusão de outras variáveis, como status nutricional e função cardíaca, que poderiam explicar o status funcional geral dos pacientes. Além disso, os resultados não podem ser generalizados para outras populações, já que foram obtidas em um centro único.

## Conclusão

A dessaturação periférica de oxigênio após a aplicação do TC6M no período pré-operatório parece ser um preditor independente do prognóstico em crianças e adolescentes submetidos à correção cirúrgica da cardiopatia congênita. A distância caminhada e as variáveis de frequência cardíaca não apresentaram a mesma associação.
