# Supplementary material for: Impacto da Capacidade Funcional Pré-operatória nos Resultados Pós-Operatórios de Cirurgia de Cardiopatia Congênita: Estudo Observacional e Prospectivo
Source: Arq Bras Cardiol. 2022 Feb 14;118(2):411–9. [Article in Portuguese] doi: 10.36660/abc.20201137 (PMC8856681; doi:10.36660/abc.20201137)
Supplement: Translation

# Translation: Impact of Preoperative Functional Capacity on Postoperative Outcomes in Congenital Heart Surgery: An Observational and Prospective Study

**Authors:** Angela Sachiko Inoue, Antônio Augusto Barbosa Lopes, Ana Cristina Sayuri Tanaka, Maria Ignêz Zanetti Feltrim, Filomena R.B.G. Galas, Juliano Pinheiro Almeida, Ludhmila Abrahão Hajjar, Emilia Nozawa

**Affiliations:** 1 Brazil Universidade de São Paulo Hospital das Clínicas, São Paulo, SP – Brazil

**Keywords:** Heart Defects, Congenital, Walk Test, Heart Rate, Physical Functional Performance, Postoperative Complications

## Abstract

**Background:**

Despite advances in surgical technique and postoperative care in congenital heart disease, cardiovascular morbidity is still high.

**Objective:**

To evaluate the association between preoperative cardiovascular fitness of children and adolescents, measured by the 6-minute walk test (6MWT) and Heart Rate Variability (HRV), and the occurrence of cardiogenic, septic shock and death in the postoperative period.

**Methods:**

Prospective, observational clinic study including 81 patients aged from 8 to 18 years. In the preoperative period, the 6MWT (distance walked and SpO_2_) and HRV were performed. The adjusted risk score for surgeries for congenital heart disease (RACHS-1) was applied to predict the surgical risk factor for mortality. The occurrence of at least one of the listed complications was considered as a combined event. P values < 0.05 were considered as significant.

**Results:**

Of the patients, 59% were male, with mean age of 12 years; 33% were cyanotic; and 72% had undergone previous cardiac surgery. Cardiogenic shock was the most common complication, and 31% had a combined event. Prior to surgery, type of current heart disease, RACHS-1, SpO_2_at rest, during the 6MWT and recovery were selected for the multivariate analysis. The SpO_2_at recovery by the 6MWT remained as an independent risk factor (OR 0.93, 95%CI [0.88 - 0.99], p=0.02) for the increasing occurrence of combined events.

**Conclusion:**

SpO_2_after the application of the 6MWT in the preoperative period was an independent predictor of prognosis in children and adolescents undergoing surgical correction; the walked distance and the HRV did not present this association.

## Introduction

In recent decades, patients with congenital heart disease have undergone more complex surgeries, and despite significant advances in surgical techniques and postoperative care, the rate of complications is still high, including cardiovascular morbidity.^1 , 2^ The possible mechanisms underlying worse postoperative outcomes in these patients seem to be the impairment of previous functional performance with reduced aerobic capacity, associated with generalized muscle weakness and autonomic nervous system disorders. Often, the presence of a residual heart defect after surgery may be partially responsible for the reduced physical capacity.^3^

To assess overall cardiovascular function, including physical capacity, exercise tests are proposed to identify risk factors for the occurrence of events in various clinical situations, such as chronic obstructive pulmonary disease^4^ and heart failure.^5^ Many tests are available to evaluate this ability, but their use in children and adolescents may provide different results from those obtained among adults due to different physiological and metabolic responses to stress.^2 , 3 , 6^

One of the methods used for the clinical assessment of aerobic fitness is the 6-minute walk test (6MWT), which is a simple test used to verify the degree of functional limitation and prognostic stratification in both adults and children.^7 , 8^ The test has been used to evaluate outcomes at different treatment stages of several conditions, and has demonstrated a strong association with oxyhemoglobin desaturation in chronic heart disease.

Abnormalities in the autonomic nervous system modulation, as measured by the heart rate variability (HRV), have been associated with increased cardiovascular mortality, with worse prognosis for heart disease and postoperative cardiac events.^9^

The objective of this study was to evaluate the association between the preoperative cardiovascular fitness status of children and adolescents, measured by the 6MWT and the HRV, and the occurrence of cardiogenic, septic shock and death in the postoperative period of congenital heart surgery.

## Methods

### Study design and patients

This was a prospective and observational clinical study conducted from January 2009 to March 2012, which included children and adolescents aged from 8 to 18 years with congenital heart disease, undergoing corrective or palliative surgical treatment at the Heart Institute of Hospital das Clínicas, Medical School of Universidade de São Paulo. This study was approved by the Research Ethics Committee of Universidade de São Paulo, Medical School, Hospital das Clínicas (number 0625/08). The informed consent was obtained for all patients from their parents or respective tutors.

The study included patients admitted for the surgical procedure who were stable. These patients were not taking inotropic or vasoactive drugs, without any potentially serious or complex arrhythmias, such as atrial / ventricular fibrillation, without implanted pacemakers, without cardiomyopathy or acquired valvular heart disease and without associated syndromes, pulmonary, neurological or orthopedic limitations. Patients whose consent was not obtained or those who did not undergo the surgical procedure were excluded.

### Data collection

The data were collected from medical records during the period of hospitalization. Once surgery was indicated, the assessment of HRV and the 6MWT were performed on the same day, in this order.

In the preoperative period, the variables age, gender, body mass index (BMI), clinical diagnosis (with greater clinical impact) and the occurrence of previous surgeries were collected. The adjusted risk score for surgeries for congenital heart disease (RACHS-1)^10^ was also applied to predict the surgical risk factor for mortality. For the use of this score, cases of congenital heart surgery are allocated to one of six risk categories, based on the presence or absence of specific diagnoses; category 1 had the lowest risk, and category 6, the highest. Regarding intraoperative and procedural variables, data were collected on the type of surgery, time of cardiopulmonary bypass (CPB), time of mechanical ventilation (MV), and use of vasoactive and / or inotropic drugs. In the postoperative period, the occurrence of death and complications until the patient’s hospital discharge was analyzed. Postoperative complications considered in the study were: death, cardiogenic shock (persistent bleeding requiring blood transfusion, need for extracorporeal membrane oxygenation (ECMO)), suspected cardiac tamponade and surgical reexploration, refractory shock, requiring inotropic support to maintain mean arterial pressure of ≥ 60mmHg (for more than 72 hours), cardiopulmonary arrest, significant arrhythmias (including atrial fibrillation, ventricular tachycardia, atrioventricular block) and septic shock (confirmation of infectious site requiring antibiotic therapy, confirmed by Infection, which develops persistent fever, respiratory failure requiring prolonged MV or use of noninvasive ventilation, persistent hypotension, and leukocytosis or leukopenia). The occurrence of at least one of the listed complications was considered as a combined event.

### 6-minute walk test

The 6-minute walk test was performed preoperatively, according to the standardized technique proposed by the American Thoracic Society^7^ in a 30-meter corridor with only one repetition. In addition to the covered distance, heart rate, oxygen saturation (SpO_2_) (Ohmeda^®^), blood pressure (Philips^®^digital sphygmomanometer), respiratory rate and subjective sensation of dyspnea and lower limb fatigue were measured using the modified Borg Rating Scale of Perceived Exertion^11^ at rest, immediately after the test, and three minutes after recovery. Standard encouragement phrases were used every minute during the test. No patient required oxygen during the test.

#### Heart rate variability

A heart rate monitor (Polar s810i^®^) was used, and electrical signals from the heart were transmitted to a monitor by an electrode strap placed around the patient’s chest. Heart rate variability (HRV) was assessed preoperatively at rest for 15 minutes, with the patient in bed at a 45 degree inclination. The first 5 minutes were used for acclimation, and the last 10 minutes, for analysis. The signal was received and sent to the Polar Precision Performance software.^12^ The artifact removal was performed with the same software, and manually, by visual inspection of R-R intervals (oscillations in the intervals between consecutive heart beats) and exclusion of abnormal intervals. Samples that presented more than 85% of sinus beats were included. The HRV analysis using linear methods was performed using Kubios HRV version 2.0 (Biosignal Analysis and Medical Imaging Group). The analyzed time domain variables were SDNN (standard deviation of normal-to-normal RR intervals recorded in a time interval); rMSSD (root mean square of the successive differences); and pNN50 (percentage of successive RR intervals that differ by more than 50 ms). The variables analyzed in the frequency domain (spectral analysis) were the high frequency (HF) component (0.15 to 0.4 Hz); the low frequency (LF) component (0.04 and 0.15Hz); the LF / HF ratio; and data normalization (n.u.) of spectral analysis to minimize the effects of other bands, such as a very low frequency component. The SDNN, rMSSD, pNN50 and HF variables were associated with parasympathetic modulation, whereas LF was associated with both sympathetic and parasympathetic modulation.^13^

#### Statistical analysis

Quantitative variables with normal distribution were presented as mean and standard deviation. Quantitative variables without normal distribution were presented as median and interquartile range (IQR; 25th and 75th percentiles). Categorical variables are presented as frequency and percentage rates. The Shapiro-Wilk test was used to evaluate the distribution of quantitative variables. The sample size calculation was based on a pilot sample in preoperative patients; for studying the prognostic through tests with 90% power and 5% significance level, the minimum prediction was of 75 cases. For the univariate analysis, regarding the combined event, the unpaired Student’s t-test or the Mann-Whitney test for quantitative variables and the chi-square or likelihood ratio test were used for categorical variables. For the multivariate analysis, variables with p<0.10 were used in the multiple logistic regression model to assess prognostic factors of death and morbidity. The probability p value <0.05 was used for the criteria of statistical significance. All analyses were performed using the SPSS 15.0 for Windows statistical package.

## Results

Ninety seven children and adolescents were evaluated, of whom 15 did not undergo surgery and one interrupted the protocol, withdrawing their consent, and therefore being excluded from the study ( Figure 1 ). Of the 81 patients in this study, 59% were male, with mean age of 12 years; 33% were cyanotic; and 72% had undergone previous heart surgery. Cardiogenic shock was the most frequent complication, and 31% had an event combined with the occurrence of at least one of the other complications. The mortality in this study was 6.2% ( Table 1 ).

**Figure 1:** – Study flowchart.

**Table 1:** – Postoperative complications of children and adolescents undergoing congenital heart surgery

Postoperative complications	n (%)
Cardiac shock	24 (29.6)
Septic shock	5 (6.2)
Death	5 (6.2)
Combined event	25 (30.9)

The factors: RACHS-1 and SpO_2_recovery were significantly associated with the occurrence of combined outcomes and are presented in Table 2 . In the postoperative period, the times of CPB, MV, ICU stay, hospital discharge and surgical procedure were significantly associated with the occurrence of combined outcomes ( Table 3 ).

**Table 2:** – Descriptive values of preoperative variables according to the group of combined event occurrence in children and adolescents undergoing congenital heart surgery

Variable		Combined event		p

	Overall	No	Yes	

	n=81	n=56 (69.1%)	n=25 (30.9%)	
Age (years)^*^	12 ± 3	12 ± 3	13 ± 2	0.190
Sex^†^				0.204
Female	31 (37.5%)	24 (42.9%)	7 (28.0%)	
Male	50 (62.5%)	32 (57.1%)	18 (72.0%)	
BMI (kg/m^2^)^Δ^	17 (12 – 27)	17(15 - 21)	17 (15 - 20)	0.581
Prior surgery^†^				0.098
No	23 (28%)	19 (33.9%)	4 (16.0%)	
Yes	58 (72%)	37 (66.1%)	21 (84.0%)	
N prior surgery^‡^				0.227
0	23 (28.40%)	19 (33.9%)	4 (16.0%)	
1	25 (30.87%)	18 (32.1%)	7 (28.0%)	
2	29 (35.81%)	17 (30.4%)	12 (48.0%)	
3	4 (4.92%)	2 (3.6%)	2 (8.0%)	
Current heart disease^Ϯ^				0.089
Non-cianotic	53 (65,43%)	40 (71.4%)	13 (52.0%)	
Cianotic	28 (34.57%)	16 (28.6%)	12 (48.0%)	
Rachs-1^‡^				0.018
1	6 (7.41%)	6 (10.8%)	0 (0.0%)	
2	22 (27.16%)	18 (32.1%)	4 (16.0%)	
3	53 (65.43%)	32 (57.1%)	21 (84.0%)	
Distance walked (meter)*	521 ± 99	517 ± 89	502 ± 94	0.480
HR rest (bpm)*	86 ± 14	87 ± 15	84 ± 10	0.326
HR 6MWT(bpm)*	127 ± 17	123 ± 20	127 ± 22	0.423
HR recovery (bpm)*	92 ± 14	92 ± 14	90 ± 11	0.372
SpO_2_rest (%)^Δ^	96 (84 – 97)	96 (85 - 98)	89 (80 - 97)	0.076
SpO_2_6MWT (%)^Δ^	94 (74 – 91)	94 (74 - 97)	83 (65 - 96)	0.050
SpO_2_recovery (%)^Δ^	96 (85 – 97)	97 (87 - 97)	87 (81 - 96)	0.009
SDNN (ms)^Δ^	37 (23 – 59)	37 (26 - 50)	33 (22 - 41)	0.184
rMSSD (ms)^Δ^	26 (13 – 46)	29 (14 - 46)	20 (14 - 28)	0.157
pNN50 (%)^Δ^	3.8 (0.2 – 26)	7.3 (0.3 – 25.6)	2.2 (0.3 – 5.8)	0.222
LF (Hz)^Δ^	402 (86 – 816)	318 (148 - 812)	312 (94 - 445)	0.225
HF (Hz)^Δ^	216 (46 – 479)	229 (39 - 526)	103 (46 - 254)	0.189
LFn.u (%)^Δ^	35 ( 50 – 79)	66 (50 - 80)	63 (53 - 80)	0.971
HFn.u (%)^Δ^	35 (21 – 55)	34 (21 - 52)	37 (20 - 47)	0.905
LF/HF^Δ^	2 (1 – 3.9)	2 (1 – 3.9)	1.7 (1.1 - 4)	0.967

*: t-Student unpaired;^†^: chi-square test;^Δ^: Mann-Whitney;^‡^: Likelihood test; n(%): number (%), or mean±standard deviation or median (interquartile range); BMI: body mass index; Rachs 1: Risk adjustment for congenital heart disease; HR: heart rate; bpm: heart beats per minute; SpO_2:_peripheral oxygen saturation; 6MWT: six-minute walk test; SDNN: standard deviation of normal-to-normal RR intervals recorded in a time interval; rMSSD: root mean square of the successive differences; pNN50: percentage of successive RR intervals that differ by more than 50 ms; LF: low frequency component ranging from 0.04 to 0.15Hz; HF: high frequency component with frequency range between 0.15 to 0.4 Hz; n. u.: normality unit.

**Table 3:** – Descriptive values of postoperative variables according to the group of occurrence of combined events in children and adolescents undergoing congenital heart surgery

Variable		Combined event		p

	Overall	No	Yes	

	n = 81	n=56 (69.1%)	n=25 (30.9%)	
Time of CPB (min)*	110 ± 70	92 ± 57	156 ± 76	<0.001
Time of MV (hours)^Δ^	11 ± 20	5.5 (3.3 - 8)	13.8 (7.8 - 19.2)	<0.001
ICU stay (day)^Δ^	8 ± 5	6 (4 - 8)	10 (7 - 16)	0.001
Hospital discharge (day)^Δ^	17 ± 12	11 (8 - 17)	21 (14 - 27)	0.001
Surgical procedure^†^				0.023
Atrial septum correction	6 (7.4%)	6 (10.7%)	0 (0.0%)	
Ventricular septum correction	7 (8.6%)	5 (8.9%)	2 (8.0%)	
RV-PA tube procedure	13 (16.1%)	7 (12.5%)	6 (24.0%)	
Fontan procedure	16 (19.8%)	9 (16.1%)	7 (28.0%)	
Mitral valve replacement	1 (1.2%)	0 (0.0%)	1 (4.0%)	
Tricuspide valve replacement	2 (2.5%)	2 (3.6%)	0 (0.0%)	
Glenn procedure	2 (2.5%)	1 (1.8%)	1 (4.0%)	
Isthmoplast	9 (11.1%)	9 (16.1%)	0 (0.0%)	
Arterial duct ligature	1 (1.2%)	1 (1.8%)	0 (0.0%)	
Pulmonary Artery Enlargement	6 (7.4%)	5 (8.9%)	1 (4.0%)	
Pulmonary valve replacement	3 (3.7%)	1 (1.8%)	2 (8.0%)	
TGA correction	2 (2.5%)	1 (1.8%)	1 (4.0%)	
Fallot tetralogy correction	2 (2.5%)	0 (0.0%)	2 (8.0%)	
Ebstein correction	3 (3.7%)	2 (3.6%)	1 (4.0%)	
Blalock-Taussig surgery	1 (1.2%)	1 (1.8%)	0 (0.0%)	
Anomalous correction of pulmonar veins	1 (1.2%)	1 (1.8%)	0 (0.0%)	
Aortic valve replacement	4 (4.9%)	4 (7.1%)	0 (0.0%)	
Others	2 (2.5%)	1 (1.8%)	1 (4.0%)	

*: t-Student unpaired;^Δ^: Mann-Whitney;^†^: chi-square test; n(%); number (%), or mean±standard deviation or median (interquartile range); CPB: cardiopulmonary bypass; MV: mechanical ventilation; ICU: intensive unit care; RV-PA: right ventricle to pulmonary artery trunk; TGA: transposition of great arteries.

When SpO_2_values were divided into groups of cyanotic and non-cyanotic heart disease, the groups showed a significant difference in relation to SpO_2_at rest, 6MWT and recovery. The non-cyanotic group has significantly higher SpO_2_values when compared to the cyanotic group ( Table 4 ).

**Table 4:** – SpO2 values in non-cyanotic and cyanotic heart disease in different moments of the 6MWT

Variable	Current heart disease		p^Δ^

	Non-cyanotic	Cyanotic	

	n=53	n=28	
SpO_2_at rest (%)^Δ^	97 (93 - 98)	78 (75 -83)	<0.001
SpO_2_6MWT (%)^Δ^	95 (96 - 97)	63 (56.5 – 68.8)	<0.001
SpO_2_recovery (%)^Δ^	97 (93 – 97.5)	80.5 (77 – 83.8)	<0.001

*^Δ^= Mann-Whitney; median (IQR).*

The variables prior surgery, types of current heart disease, RACHS-1, preoperative SpO_2_at rest, during 6MWT and recovery, and CPB time were selected to compose the multivariate analysis. The preoperative SpO_2_during recovery remained as an independent risk factor (OR 0.93, 95%CI [0.88 - 0.99], p = 0.02), for increased combined events ( Figure 2 ).

**Figure 2:** – Probabilities estimated by the logistic regression model for SpO_2_recovery.

It was observed that the higher the SpO_2_after recovery time, the lower the probability of combined events. Through the ROC curve, we observed that the cutoff point for SpO_2_is 96% (OR 3.28, 95%CI [1.21 - 8.90], p = 0.02). This point provides 68.0% sensitivity, 60.7% specificity and 63.0% accuracy. The chances of a patient with SpO_2_of less than 96% at the time of recovery to present combined events is three times higher than for patients whose SpO_2_is higher than 96%.

We observed that in the comparison between SpO_2_values in the three moments (at rest, 6MWT and recovery), in groups with and without a combined event, SpO_2_was lower, with a statistically significant difference, in the recovery period in the combined event group ( Figure 3 ).

**Figure 3:** – Box-plot with comparison of 6MWT oxygen saturation at rest, during the test and at recovery in groups with and without combined events.

## Discussion

The present study identified that the preoperative SpO_2_variable after the 6MWT was the only independent predictor of association with the occurrence of postoperative complications in children and adolescents undergoing surgical correction of congenital heart disease, and that HRV was not capable of predicting the same association. These data suggest that measuring SpO_2_may be an important tool for postoperative prognosis. By observing low preoperative SpO_2_values, clinical actions can be taken to optimize cardiorespiratory function and, thus, possibly decrease combined events in this patient population.

In children, the 6MWT was the test of choice in relation to cardiopulmonary tests on treadmills or cycle ergometers for being easy to apply, safe, and not expensive, as it does not require expensive equipment nor highly trained professionals. Studies highlight that the lack of encouragement and overprotection in this patient population have negative impacts on their physical capacity and increase the risk of developing complications over time.

The assessment of the walking distance presented lower values than those presented by Geiger et al.^14^ and Priesnitz et al.^15^ in healthy children aged more than 8 years, indicating that children with congenital heart disease have lower physical capacity than those in the healthy population. However, the present study observed that the mean walking distance was similar between the patients who presented the occurrence of a combined event and those who progressed without complications.

Monitoring pulse oximetry during the 6MWT is not a standardized procedure; however, it may offer a better estimate of gas exchange during exercise, thus showing a better correlation with the prognosis. The mechanism of oxygen desaturation may be directly related to the cardiac defect that leads to increased vascular resistance, ventricular overload, especially of the right ventricle, resulting in reduced cardiac output. Oxygen desaturation during the 6MWT is well described in patients with Chronic obstructive pulmonary disease (COPD),^4 , 16^ and interstitial lung disease,^17 , 18^ but these data, to our knowledge, are not well described among chldren to determine prognosis in the postoperative period, and this study may encourage other studies.

Schaan et al.^19^ evaluated the functional capacity of children and adolescents with congenital heart disease in a systematic review and meta-analyses, and found that the maximal oxygen consumption (VO_2max_) was the variable associated with low functional capacity, possibly being influenced by impaired chronotropic response. No measurement of pulse oximetry was reported in the presented studies.

These anatomical and pathophysiological changes may also be associated with decreased chronotropic response in this patient population. The need for reintervention is frequent and, consequently, the chances of desensitization of the β-adrenergic receptor may be directly related to altered autonomic regulation.^9 , 20^ In the present study, 72% of the patients had already undergone previous cardiac surgery; however, no statistically significant differences were observed between the patients who did or did not present with postoperative complications.

In a study by Hami et al.,^21^ in which HRV was evaluated in surgeries requiring atriotomy, the study was unable to demonstrate the influence of the surgical procedure associated with its reduction. This study observed that the Fontan procedure was the most frequent type of surgery, in 19.8% of the evaluated cases. Studies with these patients have shown a decline in physical capacity over time, attributed to reduced HRV.^22^ The use of different techniques (atriopulmonary with lateral tunnel or extracardiac tube) for this procedure does not seem to interfere, at first, with the reduction in HRV. However, the technique using the extracardiac tube seems to preserve the sinoatrial node, reducing the risk of arrhythmias. Although the fenestration technique has reduced the occurrence of postoperative complications,^23^ the reduction in SpO_2_remains a point of concern, as identified in our study.

The mortality rate in the present study was 6.2%, corroborating with other studies that showed an incidence between 3.6% and 15%. Approximately 66% of the patients were classified in Category 3 of RACHS-1, which, according to Jenkins et al.,^10^ has a mortality rate of around 9.5%, confirming the complexity of heart diseases. All deaths that occurred in the ICU evolved from postoperative heart failure.

Finally, in this study, other variables were associated with postoperative complications. We identified that times of CPB, MV, ICU stay, hospital discharge and surgical procedure were significantly associated with the occurrence of combined outcomes. Giamberti et al.^24^ described that severe morbidity is relatively frequent, and generally associated with the preoperative (high level of hematocrits due to cyanosis, congestive heart failure, and the number of previous operations) and operative (Fontan procedure/conversion and cardiopulmonary bypass duration) conditions of the patient. In fact, 84% of the patients with complications had undergone previous surgery and had more time of CPB, MV and length of hospital stay.

Our study has some potential limitations, such as including a heterogeneous sample and the non-inclusion of other variables, like nutritional status and cardiac function, which could explain the overall functional status of patients. In addition, the results cannot be generalized to other populations, since they were obtained at a single center.

## Conclusion

In conclusion, the peripheral oxygen desaturation after the application of the 6MWT in the preoperative period seems to be an independent predictor of prognosis in children and adolescents undergoing surgical correction of congenital heart disease. The walked distance and the variables of heart rate variability did not present the same association.
